# (*E*)-1-(3-Chloro­phen­yl)-2-(2-oxidonaphthalen-1-yl)diazen-1-ium

**DOI:** 10.1107/S1600536813014931

**Published:** 2013-06-08

**Authors:** Ali Benosmane, Assia Mili, Hassiba Bouguerria, Abdelkader Bouchoul

**Affiliations:** a Unité de recherche de Chimie de l’Environnement et Moléculaire Structurale, Faculté du sciences exactes, Université Mentouri de Constantine 1, 25000 Constantine, Algeria

## Abstract

The title zwitterion,, C_16_H_11_ClN_2_O, is approximately planar, the dihedral angle between the benzene ring and naphthalene ring system is 1.55 (13)°; an intra­molecular N—H⋯O hydrogen bond stabilizes the planar conformation. In the crystal, π–π stacking between the benzene ring and the naphthalene ring system of adjacent mol­ecules links the mol­ecules into supra­molecular chains running along the *b* axis, the centroid–centroid distance being 3.765 (2) Å.

## Related literature

For general background to the use of azo compounds as dyes, pigments and advanced materials, see: Lee *et al.* (2004 ▶); Oueslati *et al.* (2004 ▶). Many azo compounds have been synthesized by diazo­tization and diazo-coupling reactions; for information, see: Wang *et al.* (2003 ▶). For a related structure, see: Elmali *et al.* (2001 ▶).
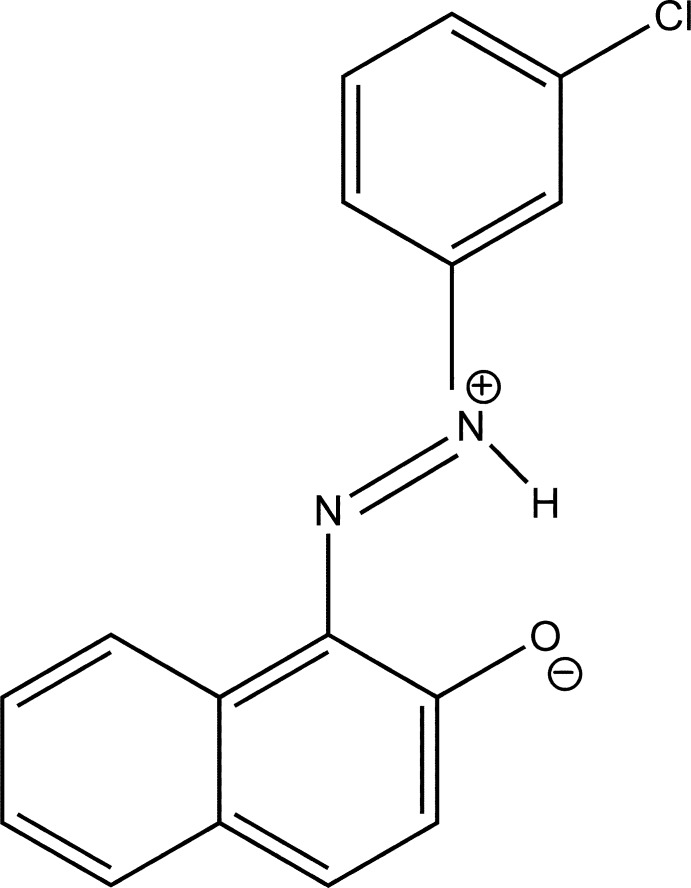


## Experimental

### 

#### Crystal data

C_16_H_11_ClN_2_O
*M* *_r_* = 282.72
Monoclinic, 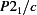

*a* = 16.340 (2) Å
*b* = 5.7665 (4) Å
*c* = 15.632 (2) Å
β = 113.604 (4)°
*V* = 1349.7 (3) Å^3^
*Z* = 4
Mo *K*α radiation
μ = 0.28 mm^−1^
*T* = 293 K
0.09 × 0.04 × 0.02 mm

#### Data collection

Nonius KappaCCD diffractometer
4488 measured reflections
2418 independent reflections
1289 reflections with *I* > 2σ(*I*)
*R*_int_ = 0.042

#### Refinement

*R*[*F* ^2^ > 2σ(*F* ^2^)] = 0.059
*wR*(*F* ^2^) = 0.185
*S* = 1.01
2418 reflections
171 parameters
H-atom parameters constrained
Δρ_max_ = 0.31 e Å^−3^
Δρ_min_ = −0.28 e Å^−3^

### 

Data collection: *KappaCCD Reference Manual* (Nonius, 1998 ▶); cell refinement: *DENZO* and *SCALEPACK* (Otwinowski & Minor, 1997 ▶); data reduction: *DENZO* and *SCALEPACK*; program(s) used to solve structure: *SHELXS97* (Sheldrick, 2008 ▶); program(s) used to refine structure: *SHELXL97* (Sheldrick, 2008 ▶); molecular graphics: *ORTEP-3 for Windows* (Farrugia, 2012 ▶); software used to prepare material for publication: *SHELXL97*.

## Supplementary Material

Crystal structure: contains datablock(s) global, I. DOI: 10.1107/S1600536813014931/xu5708sup1.cif

Structure factors: contains datablock(s) I. DOI: 10.1107/S1600536813014931/xu5708Isup2.hkl

Supplementary material file. DOI: 10.1107/S1600536813014931/xu5708Isup3.cml

Additional supplementary materials:  crystallographic information; 3D view; checkCIF report

## Figures and Tables

**Table 1:** Hydrogen-bond geometry (Å, °)

*D*—H⋯*A*	*D*—H	H⋯*A*	*D*⋯*A*	*D*—H⋯*A*
N1—H1⋯O1	0.94	1.82	2.564 (4)	135

**Fig. 1.**
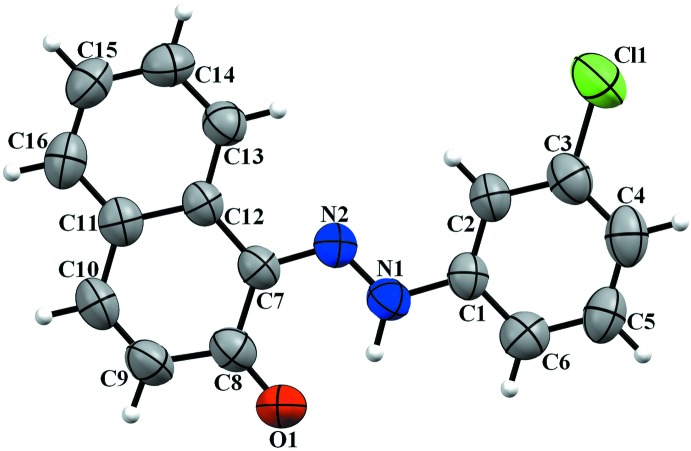
The molecular structure.

## supplementary crystallographic information

### Comment

Azo-compounds are very important in the fields of dyes, pigments and advanced materials (Lee *et al.*, 2004; Oueslati *et al.*, 2004). Azo-dyes are synthetic pigments that contain an azo-group, as part of the structure. Azo-groups do not occur naturally. Many azo-compounds have been synthesized by the diazotization and diazo coupling reaction (Wang *et al.*, 2003). The title compound was obtained through the diazotization of 3-chloroaniline followed by a coupling reaction with 2-naphthol.

This compound, which has a non-planar molecular structure, contains two aromatic rings linked through a imine group. The dihedral angle between the two aromatic rings C1—C6 and C7—C16 is 1.55 (13)°. Intramolecular N—H···O hydrogen bond is observed in the molecular structure, similar to that in a reported structure (Elmali *et al.*, 2001). In the crystal structure, molecules are linked through π-π stacking between benzene ring and naphthalene ring system of adjacent molecules, the centroid-centroid distance between C1-ring and C7^i^-ring being 3.765 (2) Å (symmetry code: i = x, -1+y, z).

### Experimental

The title compound was obtained through the diazotization of 3-chloroaniline followed by a coupling reaction with 2-naphthol. Crystals suitable for X-ray analysis were obtained by slow evaporation of a pentane solution.

### Refinement

H atoms were positioned geometrically with C—H = 0.93 and N—H = 0.94 Å, and refined as riding with *U*_iso_(H) = 1.2*U*_eq_(carrier).

### Crystal data

**Table:**

C_16_H_11_ClN_2_O	*F*(000) = 584
*M**_r_* = 282.72	*D*_x_ = 1.391 Mg m^−^^3^
Monoclinic, *P*2_1_/*c*	Mo *K*α radiation, λ = 0.71073 Å
Hall symbol: -P 2ybc	Cell parameters from 2548 reflections
*a* = 16.340 (2) Å	θ = 2.9–25.4°
*b* = 5.7665 (4) Å	µ = 0.28 mm^−^^1^
*c* = 15.632 (2) Å	*T* = 293 K
β = 113.604 (4)°	Needle, red
*V* = 1349.7 (3) Å^3^	0.09 × 0.04 × 0.02 mm
*Z* = 4	

### Data collection

**Table:**

Nonius KappaCCD diffractometer	1289 reflections with *I* > 2σ(*I*)
Radiation source: fine-focus sealed tube	*R*_int_ = 0.042
Horizonally mounted graphite crystal monochromator	θ_max_ = 25.3°, θ_min_ = 3.1°
Detector resolution: 9 pixels mm^-1^	*h* = −19→19
CCD rotation images, thick slices scans	*k* = −6→6
4488 measured reflections	*l* = −18→18
2418 independent reflections	

### Refinement

**Table:**

Refinement on *F*^2^	Primary atom site location: structure-invariant direct methods
Least-squares matrix: full	Secondary atom site location: difference Fourier map
*R*[*F*^2^ > 2σ(*F*^2^)] = 0.059	Hydrogen site location: inferred from neighbouring sites
*wR*(*F*^2^) = 0.185	H-atom parameters constrained
*S* = 1.01	*w* = 1/[σ^2^(*F*_o_^2^) + (0.0928*P*)^2^ + 0.1732*P*] where *P* = (*F*_o_^2^ + 2*F*_c_^2^)/3
2418 reflections	(Δ/σ)_max_ < 0.001
171 parameters	Δρ_max_ = 0.31 e Å^−^^3^
0 restraints	Δρ_min_ = −0.28 e Å^−^^3^

### Special details

**Table:**

Geometry. Bond distances, angles *etc*. have been calculated using the rounded fractional coordinates. All su's are estimated from the variances of the (full) variance-covariance matrix. The cell e.s.d.'s are taken into account in the estimation of distances, angles and torsion angles
Refinement. Refinement of *F*^2^ against ALL reflections. The weighted *R*-factor *wR* and goodness of fit *S* are based on *F*^2^, conventional *R*-factors *R* are based on *F*, with *F* set to zero for negative *F*^2^. The threshold expression of *F*^2^ > σ(*F*^2^) is used only for calculating *R*-factors(gt) *etc*. and is not relevant to the choice of reflections for refinement. *R*-factors based on *F*^2^ are statistically about twice as large as those based on *F*, and *R*- factors based on ALL data will be even larger.

### Fractional atomic coordinates and isotropic or equivalent isotropic displacement parameters (Å^2^)

**Table:**

	*x*	*y*	*z*	*U*_iso_*/*U*_eq_	
Cl1	0.06364 (8)	−0.5972 (2)	0.37478 (9)	0.1078 (5)	
O1	0.46478 (16)	0.1141 (4)	0.38441 (18)	0.0786 (9)	
N1	0.33259 (17)	−0.1220 (4)	0.38634 (18)	0.0572 (9)	
N2	0.27805 (17)	0.0115 (4)	0.32242 (17)	0.0528 (8)	
C1	0.2977 (2)	−0.3084 (5)	0.4184 (2)	0.0535 (10)	
C2	0.2070 (2)	−0.3500 (5)	0.3853 (2)	0.0583 (11)	
C3	0.1771 (2)	−0.5415 (6)	0.4175 (2)	0.0667 (12)	
C4	0.2351 (3)	−0.6891 (6)	0.4821 (3)	0.0746 (14)	
C5	0.3258 (3)	−0.6464 (6)	0.5165 (2)	0.0708 (14)	
C6	0.3578 (2)	−0.4547 (6)	0.4852 (2)	0.0666 (12)	
C7	0.3117 (2)	0.1901 (5)	0.2907 (2)	0.0523 (11)	
C8	0.4066 (2)	0.2395 (5)	0.3238 (2)	0.0613 (11)	
C9	0.4306 (2)	0.4390 (6)	0.2841 (3)	0.0714 (14)	
C10	0.3705 (3)	0.5765 (6)	0.2221 (3)	0.0670 (12)	
C11	0.2759 (2)	0.5349 (5)	0.1888 (2)	0.0572 (11)	
C12	0.2463 (2)	0.3361 (5)	0.2220 (2)	0.0504 (10)	
C13	0.1542 (2)	0.2970 (5)	0.1879 (2)	0.0618 (11)	
C14	0.0948 (2)	0.4492 (6)	0.1263 (3)	0.0730 (12)	
C15	0.1241 (3)	0.6417 (6)	0.0963 (2)	0.0698 (14)	
C16	0.2134 (3)	0.6844 (5)	0.1264 (2)	0.0684 (13)	
H1	0.39440	−0.10220	0.40590	0.0690*	
H2	0.16670	−0.25050	0.34200	0.0700*	
H4	0.21350	−0.81740	0.50270	0.0900*	
H5	0.36550	−0.74560	0.56060	0.0850*	
H6	0.41880	−0.42450	0.50860	0.0800*	
H9	0.49090	0.47380	0.30250	0.0860*	
H10	0.39040	0.70430	0.19950	0.0800*	
H13	0.13290	0.16620	0.20720	0.0740*	
H14	0.03380	0.42060	0.10480	0.0870*	
H15	0.08320	0.74400	0.05520	0.0840*	
H16	0.23270	0.81510	0.10510	0.0820*	

### Atomic displacement parameters (Å^2^)

**Table:**

	*U*^11^	*U*^22^	*U*^33^	*U*^12^	*U*^13^	*U*^23^
Cl1	0.0856 (8)	0.1168 (9)	0.1281 (10)	−0.0275 (6)	0.0501 (7)	0.0075 (7)
O1	0.0545 (15)	0.0780 (15)	0.0954 (19)	0.0021 (12)	0.0218 (14)	0.0107 (14)
N1	0.0531 (16)	0.0556 (14)	0.0611 (17)	−0.0055 (13)	0.0212 (14)	−0.0046 (14)
N2	0.0587 (16)	0.0505 (13)	0.0489 (15)	−0.0023 (12)	0.0213 (13)	−0.0068 (12)
C1	0.066 (2)	0.0448 (16)	0.0501 (18)	−0.0040 (14)	0.0237 (17)	−0.0070 (14)
C2	0.067 (2)	0.0518 (17)	0.059 (2)	−0.0067 (16)	0.0284 (17)	−0.0001 (16)
C3	0.076 (2)	0.068 (2)	0.063 (2)	−0.0122 (18)	0.035 (2)	−0.0072 (18)
C4	0.111 (3)	0.056 (2)	0.065 (2)	−0.010 (2)	0.044 (2)	−0.0048 (18)
C5	0.096 (3)	0.058 (2)	0.056 (2)	0.0043 (19)	0.028 (2)	0.0039 (17)
C6	0.072 (2)	0.066 (2)	0.058 (2)	−0.0013 (17)	0.0222 (18)	−0.0094 (17)
C7	0.055 (2)	0.0517 (17)	0.0529 (18)	−0.0054 (14)	0.0244 (16)	−0.0074 (15)
C8	0.059 (2)	0.0605 (19)	0.067 (2)	−0.0076 (17)	0.0281 (18)	−0.0088 (17)
C9	0.058 (2)	0.077 (2)	0.086 (3)	−0.0100 (19)	0.036 (2)	−0.003 (2)
C10	0.076 (2)	0.063 (2)	0.071 (2)	−0.0154 (18)	0.039 (2)	−0.0017 (17)
C11	0.070 (2)	0.0540 (17)	0.0536 (19)	−0.0028 (16)	0.0310 (17)	−0.0088 (15)
C12	0.0576 (19)	0.0468 (16)	0.0502 (18)	−0.0044 (14)	0.0251 (15)	−0.0082 (14)
C13	0.060 (2)	0.0612 (19)	0.062 (2)	−0.0075 (16)	0.0220 (18)	−0.0024 (17)
C14	0.058 (2)	0.082 (2)	0.069 (2)	0.0026 (19)	0.0149 (18)	−0.003 (2)
C15	0.078 (3)	0.063 (2)	0.059 (2)	0.0111 (19)	0.0177 (19)	0.0035 (17)
C16	0.097 (3)	0.0522 (19)	0.057 (2)	0.0012 (18)	0.032 (2)	0.0015 (17)

### Geometric parameters (Å, º)

**Table:**

Cl1—C3	1.731 (4)	C11—C16	1.393 (5)
O1—C8	1.266 (4)	C11—C12	1.421 (4)
N1—N2	1.293 (4)	C12—C13	1.399 (5)
N1—C1	1.400 (4)	C13—C14	1.375 (5)
N2—C7	1.352 (4)	C14—C15	1.364 (5)
N1—H1	0.9400	C15—C16	1.364 (7)
C1—C2	1.381 (5)	C2—H2	0.9300
C1—C6	1.395 (4)	C4—H4	0.9300
C2—C3	1.382 (5)	C5—H5	0.9300
C3—C4	1.370 (5)	C6—H6	0.9300
C4—C5	1.381 (7)	C9—H9	0.9300
C5—C6	1.393 (5)	C10—H10	0.9300
C7—C8	1.453 (5)	C13—H13	0.9300
C7—C12	1.444 (4)	C14—H14	0.9300
C8—C9	1.434 (5)	C15—H15	0.9300
C9—C10	1.331 (6)	C16—H16	0.9300
C10—C11	1.440 (6)		
			
N2—N1—C1	118.7 (3)	C7—C12—C11	118.9 (3)
N1—N2—C7	118.8 (3)	C7—C12—C13	123.5 (3)
C1—N1—H1	121.00	C12—C13—C14	120.9 (3)
N2—N1—H1	120.00	C13—C14—C15	120.9 (4)
N1—C1—C6	117.8 (3)	C14—C15—C16	120.2 (3)
C2—C1—C6	120.2 (3)	C11—C16—C15	120.9 (3)
N1—C1—C2	121.9 (3)	C1—C2—H2	120.00
C1—C2—C3	119.0 (3)	C3—C2—H2	121.00
Cl1—C3—C4	119.2 (3)	C3—C4—H4	120.00
Cl1—C3—C2	119.2 (3)	C5—C4—H4	120.00
C2—C3—C4	121.6 (3)	C4—C5—H5	120.00
C3—C4—C5	119.6 (4)	C6—C5—H5	120.00
C4—C5—C6	120.0 (3)	C1—C6—H6	120.00
C1—C6—C5	119.5 (3)	C5—C6—H6	120.00
C8—C7—C12	121.1 (3)	C8—C9—H9	119.00
N2—C7—C8	123.5 (3)	C10—C9—H9	119.00
N2—C7—C12	115.4 (3)	C9—C10—H10	119.00
C7—C8—C9	116.2 (3)	C11—C10—H10	119.00
O1—C8—C9	121.9 (3)	C12—C13—H13	119.00
O1—C8—C7	121.9 (3)	C14—C13—H13	120.00
C8—C9—C10	122.9 (4)	C13—C14—H14	120.00
C9—C10—C11	122.4 (4)	C15—C14—H14	120.00
C12—C11—C16	119.6 (3)	C14—C15—H15	120.00
C10—C11—C12	118.4 (3)	C16—C15—H15	120.00
C10—C11—C16	122.0 (3)	C11—C16—H16	120.00
C11—C12—C13	117.6 (3)	C15—C16—H16	120.00
			
C1—N1—N2—C7	−179.3 (3)	N2—C7—C12—C13	−1.3 (4)
N2—N1—C1—C2	−1.5 (4)	C8—C7—C12—C11	−1.9 (4)
N2—N1—C1—C6	178.0 (3)	C8—C7—C12—C13	−179.8 (3)
N1—N2—C7—C8	0.8 (4)	O1—C8—C9—C10	−178.3 (4)
N1—N2—C7—C12	−177.7 (3)	C7—C8—C9—C10	1.5 (5)
N1—C1—C2—C3	177.8 (3)	C8—C9—C10—C11	−0.7 (6)
C6—C1—C2—C3	−1.7 (5)	C9—C10—C11—C12	−1.6 (6)
N1—C1—C6—C5	−177.9 (3)	C9—C10—C11—C16	177.7 (4)
C2—C1—C6—C5	1.6 (5)	C10—C11—C12—C7	2.8 (4)
C1—C2—C3—Cl1	−178.9 (2)	C10—C11—C12—C13	−179.2 (3)
C1—C2—C3—C4	0.8 (5)	C16—C11—C12—C7	−176.5 (3)
Cl1—C3—C4—C5	179.9 (3)	C16—C11—C12—C13	1.5 (4)
C2—C3—C4—C5	0.2 (6)	C10—C11—C16—C15	−179.6 (3)
C3—C4—C5—C6	−0.3 (6)	C12—C11—C16—C15	−0.4 (5)
C4—C5—C6—C1	−0.7 (5)	C7—C12—C13—C14	176.4 (3)
N2—C7—C8—O1	1.1 (5)	C11—C12—C13—C14	−1.6 (5)
N2—C7—C8—C9	−178.7 (3)	C12—C13—C14—C15	0.4 (5)
C12—C7—C8—O1	179.6 (3)	C13—C14—C15—C16	0.8 (6)
C12—C7—C8—C9	−0.2 (4)	C14—C15—C16—C11	−0.8 (5)
N2—C7—C12—C11	176.7 (3)		

### Hydrogen-bond geometry (Å, º)

**Table:**

*D*—H···*A*	*D*—H	H···*A*	*D*···*A*	*D*—H···*A*
N1—H1···O1	0.94	1.82	2.564 (4)	135
